# New mental health laws in bosnia and herzegovina- step towards in our practice

**DOI:** 10.1192/j.eurpsy.2021.1064

**Published:** 2021-08-13

**Authors:** G. Racetovic, M. Latinovic, G. Cerkez, E. Mesic

**Affiliations:** 1 Community Mental Health Center, Health Center Prijedor, Prijedor, Bosnia and Herzegovina; 2 Department For Health Care, Ministry of Health and Social Welfare of the Republic of Srpska, Banja Luka, Bosnia and Herzegovina; 3 Department For Public Health, Federal Ministry of Health, Sarajevo, Bosnia and Herzegovina; 4 Project Of Mental Health In Bosnia And Herzegovina, Association XY, Sarajevo, Bosnia and Herzegovina

**Keywords:** Mental Health, Law, Stigma

## Abstract

**Introduction:**

Establishing broad spectrum of new mental health services in whole Bosnia and Herzegovina (BH) existing mental health laws in both entities needed to be upgrade according positive results of the mental health reform in the country. Previous laws were exclusivelly oriented on protection of rights of the people with mental health disorders in (mainly) psychiatric institutions and were progressive and new in the period of their implementation (2001-2004).

**Objectives:**

Since 2010 main reform processes had direction to community mental health care and developed positive movement with implementing new services oriented to patients and their needs/continuity of care. For example case management and occupational therapy are part of daily work in whole country and standards established trough accreditation process lead to uniform approach in community work in the area of mental health.

**Methods:**

Comparative analysis of laws in BH concerning mental health.

**Results:**

Carefuly and good preparation for pronounce of new Mental Health Law both in Republic of Srpska and Federation of BH were supported from both entities (task forces, drafts and proposition of the law and public discussion) and they are formaly supported in both entities parliaments in 2020 (prolonged since COVID-19 situation). In both laws is more emphasised role of commnity services, prevention, and post-hospital rehabilitation as continuity of care. Book of rules that follow the laws will be establish no longer than the end of 2020.

**Conclusions:**

New mental health laws in BH are path to better protection of mental health of all population in BH and rights of our patients’ recovery.

